# Modeling the Voltage Produced by Ultrasound in Seawater by Stochastic and Artificial Intelligence Methods †

**DOI:** 10.3390/s22031089

**Published:** 2022-01-30

**Authors:** Alina Bărbulescu, Cristian Ștefan Dumitriu

**Affiliations:** 1 Department of Civil Engineering, Transylvania University of Brașov, 5 Turnului Str., 900152 Brasov, Romania; alina.barbulescu@unitbv.ro; 2 Department of Installations for Constructions, Transylvania University of Brașov, 5 Turnului Str., 900152 Brasov, Romania

**Keywords:** cavitation, voltage, Generalized Regression Neural Network (GRNN), autoregressive integrated moving average (ARIMA), Wavelet-ARIMA, wavelet-artificial neural network (ANN)

## Abstract

Experiments have proved that an electrical signal appears in the ultrasonic cavitation field; its properties are influenced by the ultrasound frequency, the liquid type, and liquid characteristics such as density, viscosity, and surface tension. Still, the features of the signals are not entirely known. Therefore, we present the results on modeling the voltage collected in seawater, in ultrasound cavitation produced by a 20 kHz frequency generator, working at 80 W. Comparisons of the Box–Jenkins approaches, with artificial intelligence methods (GRNN) and hybrid (Wavelet-ARIMA and Wavelet-ANN) are provided, using different goodness of fit indicators. It is shown that the last approach gave the best model.

## 1. Introduction

Cavitation is the process of apparition, augmentation, and collapse of the bubbles created around tiny particles in special pressure conditions in a liquid [1]. The discontinuity of the liquids’ state characterizes this phenomenon when the pressure experiences a sudden local drop [2]. The cavitation that appears at the ultrasound propagation in fluids is named ultrasound cavitation [3,4,5].

Effects as corrosion–erosion [6,7,8], the apparition of vibration and noise [9,10,11], sonoluminescence [12], and solid materials’ unpassivation are associated with the ultrasonic cavitation. Ultrasonic cavitation is employed for bleaching, soldering, emulsification, cleaning, extraction, nanoparticles synthesis, and separation [3,9].

Different researchers have analyzed acoustic cavitation and the phenomena associated with sound propagation in liquids for the last twenty years. Still, the physical processes related to the formation and the bubbles’ collapse are not entirely explained. They result from the interaction of the bubbles whose increasing-collapse cycles are not simultaneous, giving birth to the multiple interactions [13,14].

Industrial processes (among which ultrasonic cleaning and sonochemical processing) depend on the acoustic cavitation, which at its turn, is influenced by the conditions of the ultrasonic process [15]. Last period, increasing interest in understanding the ultrasound cavitation mechanism and explaining its effects was noticed [11,12,16,17,18].

Scientists presented applications of acoustic cavitation. They are based on the analysis of the signal spectrum that appeared in the cavitation field [19]. It has been proved that the signal generated by exciting the transducer by a high frequency (in this case, 20 kHz) depends on the liquid nature and the power regime. The electrical signal is alternative as a result of the cyclical augmentation–explosion process suffered by the bubbles [11,16,17,20].

Other researchers focused on eliminating the noise produced in acoustic cavitation [17] and explained the apparition of the electrical signal in cavitating liquids using the theory of double layer from the physics of plasma [16,20]. Differential equations, ARIMA, and artificial neural networks were proposed for characterizing the signal in the time domain [3,19,21,22].

Despite the good performances of the Box–Jenkins approaches on modeling such signals, they cannot capture the nonlinear behavior of the time series. Artificial intelligence (AI) methods have come to fill a gap in this direction. Generalized Regression Neural Networks (GRNN) proved their capabilities in modeling in domains such as medicine, IoT, agriculture, meteorology, and finances [23,24,25,26,27,28]. Although it requires a longer time to provide the results by comparison to other algorithms, GRNN [29] have some capabilities leading many to recommend their use: they learn quickly, do not use backpropagation, are not sensitive to the noise, and can be modified to permit a multidimensional output [29,30].

Many scientists, among which Anjoy and Paul [31], Fard and Akbari-Zadehb [32], Khandelwal et al. [33], Lopes et al. [34], Wang et al. [35] showed that the hybrid approaches often provide better models that the Box–Jenkins or AI models, separately, capturing both the linear and nonlinear behavior.

In the above context, the main contributions to the knowledge in the field are the following. (1) Building an experimental installation for the study of ultrasound cavitation in different liquids. (2) Collecting the electrical signals induced by cavitation in seawater. (3) Studying the statistical properties of these signals. (4) Proposing four alternative approaches—ARIMA, Generalized Regression Neural Network (GRNN), Wavelet-ARIMA, and Wavelet-ANN (the last two being hybrid)—for modeling the collected signals in seawater. (5) Using the built models for forecasting the series. (6) Comparing the performances of these approaches based on the forecast quality.

## 2. Materials and Methods

### 2.1. Experiment

**Figure 1:**
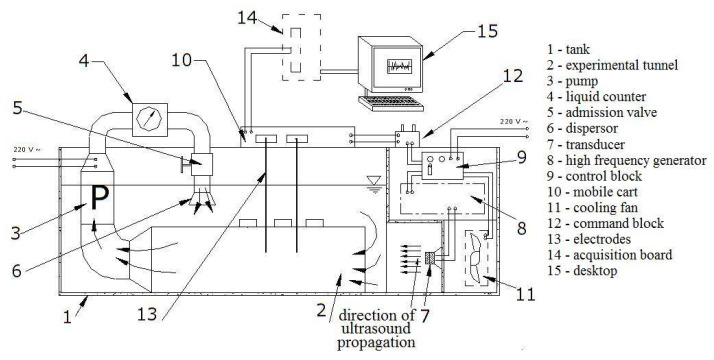
The experimental setup [20].

The installation used for this experiment was built for the study in stationary and circulating liquids. It has the following main components (in the brackets, we refer to the numbers from Figure 1) [20]:

- The tank where the studied liquid (seawater, here) is introduced (1).
- The generator of high frequency (HAMEG HM8130, Germany), that worked at 20 kHz in this case (8).
- A ceramic transducer (7), excited by the generator.
- A pair of electrodes made of copper (13), used to collect the electrical signal produced when the generator works. They can be placed at various distances (from 6 to 61 cm) from the transducer. In the experiment presented here, the distance transducer-electrodes was 30 cm.
- An acquisition board (14), connected to a computer (15) for recording the signal.
- A cooling fan (11), utilized for preserving a constant temperature of the liquid (in this case 20 °C) during the experiment (given that the ultrasound cavitation is an exogenous phenomenon).
- The command block (12), used for selecting different powers for the generator regime (80 W, 120 W, or 180 W). Here, 80 W was selected.

When the experiment is performed in stationary liquid, the other components of the experimental setup are not involved. Details on the setup functioning are given in [20,22].

**Figure 2:**
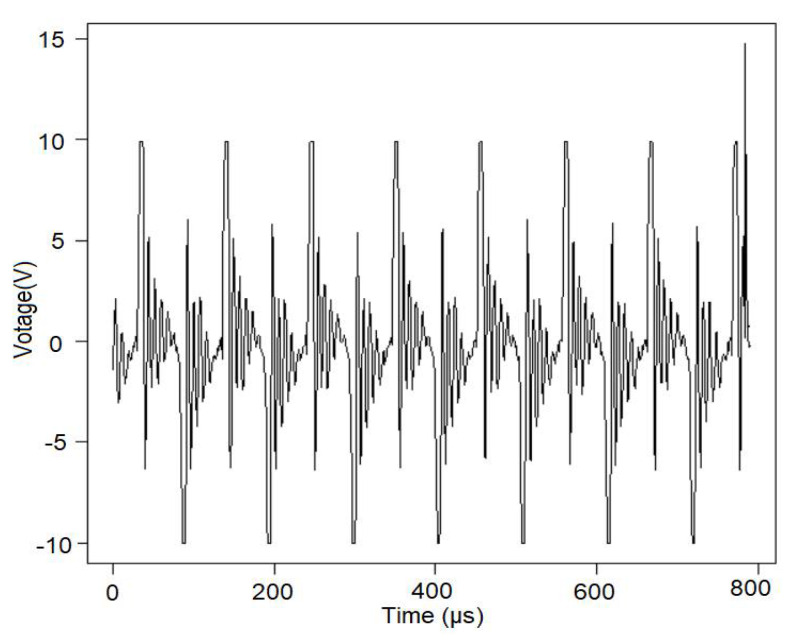
Voltage collected at 80 W in seawater (built using the R software).

The studied signal is represented in Figure 2.

### 2.2. Methodology

The signal, studied in the time domain, was subject to statistical tests performed at the significance level of 5%. The null hypotheses, the alternatives, and the tests performed are as follows:

- Normality, against non-normality—the Shapiro–Wilk test [36].
- Homoskedasticity, against heteroskedasticity—the Levene test [37].
- Randomness, against non-randomness—the runs test [38].
- Stationarity against non-stationarity—KPSS test [39].

The statistical analysis and mathematical modeling were performed in Minitab17, DTREG, and R software (version 4.0.5).

#### 2.2.1. ARMA Models

A discrete-time process is a sequence of real random variables, (*X_t_*; *t*∈Z).

A time process (*X_t_*; *t*) is called stationary if it satisfies the following conditions:

- $\forall t\in Z,\;M\left(X_{t}^{2}\right)<+\infty ,$
- $\forall t\in Z,\;M\left(X_{t}\right)<+\infty$ and is invariant in time (M denotes the expectation),
- $\forall t,h\in Z,\;\mathrm{Cov}\left(X_{t},\;X_{t+h}\right)=\gamma \left(h\right)$ (i.e., the covariance of $X_{t}$ and $X_{t+h}$ depends only on the lag *h*).

An ARMA(*p*,*q*) is a model whose equation is

(1) $$X_{t}=c+\varepsilon_{t}+\sum_{i=1}^{p}\varphi_{i}X_{t-i}+\overset{q}{\underset{j=1}{\sum }}\theta_{j}\varepsilon_{t-j},\;\varphi_{p}\neq 0,\theta_{q}\;\neq 0,\;$$

where ($\varepsilon_{t};t\in Z$) {\displaystyle \varepsilon _{t}} are Gaussian, independent random variables with the same variance and the mean equal to zero.

The first *p* terms are autoregressive, and the last *q*—moving-average.

Given a data series, the model selection depends on the series stationarity or non-stationarity. If a series is not stationary, its stationarity can be reached by taking a difference of the *d*-th order of the series terms ($d\in N^{*})$.

ARMA(*p*,*q*) is a particular type of autoregressive integrated moving average processes, ARIMA(*p*,*d*,*q*) [40]. The degree of differentiation, *d*, in ARIMA(*p,d,q*) processes is chosen for reaching the series stationarity. If the series is stationary, then *d* = 0, and the model is of ARIMA(*p*,0,*d*) = ARMA(*p,q*) type [40].

The chart of the series autocorrelation function provides information on selecting an ARMA(*p*,*q*). If *p* > *q*, the values of the autocorrelation function belong to a curve formed by a mixture of exponential decreasing and damped sinusoid functions.

The model validation is done by applying the *t*-test to the model’s coefficients and testing the hypotheses on ($\varepsilon_{t};t\in Z$) {\displaystyle \varepsilon _{t}} (that should form a white noise). The selection of the best model among the ARMA competitors that fulfill the statistical tests on the coefficients and residual ($\varepsilon_{t};\;t\in Z$) {\displaystyle \varepsilon _{t}} is made based on the Akaike (AIC) or Schwarz (SCH) criteria {\displaystyle \varepsilon _{t}} [40]. The lowest the AIC (SCH) is, the better the model is {\displaystyle \varepsilon _{t}}.

#### 2.2.2. Generalized Regression Neural Networks

**Figure 3:**
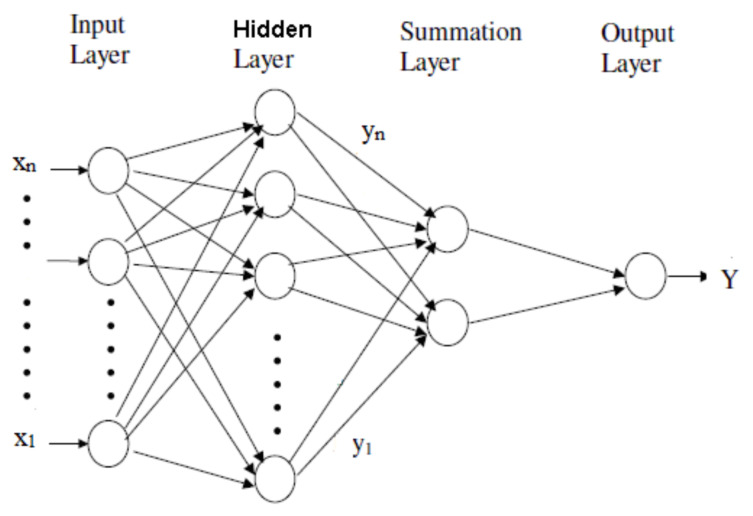
GRNN diagram.

Generalized Regression Neural Network belongs to the group probabilistic neural networks. The GRNN architecture is presented in Figure 3. It contains four layers—Input, Hidden, Summation, and Output [29]. Given its ability to capture nonlinearities, learning without backpropagation, and the use of nonparametric regression, GRNN was widely utilized for solving classification, regression, and forecast problems that involve continuous variables [29,30].

The Input layer is formed of the vector of the recorded values *X* = ($x_{1},\;\ldots ,\;x_{n})\;($Figure 3), in this study, the signal’s values.

The neurons in the hidden layer apply a kernel function to the distances between the training data and the prediction point. The σ values are used for estimating the influence radius. The best σ should be determined when training the network to control the distributions of the kernel function [29]. The most used approach for finding the optimum σ is minimizing the mean square error (MSE). This study employed the conjugate gradient algorithm to estimate the best σ for the entire model.

The RBF and reciprocal kernels have been utilized in this research. As no significant improvement was noticed in the second case, we report the results obtained using the RBF kernel.

After training, the number of neurons in the hidden layer is the same as the number of training samples involved in the modeling [29]. The hidden layer also stores all the variables’ values.

The summation layer consists of the D- and S-summation neurons that collect the information from all the neurons from the previous layer. Both neurons sum the values from the hidden layer. The sum is weighted (with the sum of weights equal to 1) in the D-summation neuron [29].

The output layer contains the results of dividing the values stored in each neuron from the previous layer, providing the most probable value for the dependent variable, Y.

An optimization process may also be performed to remove unnecessary neurons. In this case, the optimization criterion was error minimization.

To perform the modeling, the series was divided into a ratio training: test = 80:20 and 70:30. As the best results have been obtained using the first partition, we present this result. The number of iterations was fixed to 5000 (maximum) and 1000 (without improvement). The values of σ were searched in the interval 0.0001–10.

#### 2.2.3. Wavelet-ARIMA Model

A discrete wavelet transform (DWT) is a used to decompose a signal into several sets; each of them is a time series of coefficients that describes the evolution of the signal in time in the corresponding frequency band [41].

The discrete wavelet transform, usually utilized for modeling purposes, is not invariant to translation. To surpass this drawback, the non-decimated wavelet transform (NDWT) may be employed [42].

Considering the signal *X* = (*X*_1, …,_ *X_N_*), its decomposition by the à trous wavelet transform results in

(2) $$X_{t}=c_{J,t}+\overset{J}{\underset{j=1}{\sum }}w_{j,t}$$

where the first term represents a smooth version of the initial signal, and the term under the sum is the signal’s ‘detail’ at the scale $2^{-j}$ [43,44].

In this article, we used the Wavelet-ARIMA algorithm proposed in [43] and developed by Aminghafari and Poggi [45].

The non-decimated Haar algorithm employs the filter *h* = (1/2, 1/2), leading to the following equation for the reconstruction of the value $X_{N+1}$ is

(3) $$X_{N+1}=c_{J,N+1}+\overset{J}{\underset{j=1}{\sum }}w_{j,N+1}$$

where $c_{J,N+1}$ and $w_{j,N+1}$ are the approximation and detail coefficients in the NDWT.

Thus, for predicting $X_{N+1}$, it is necessary to estimate the non-decimated (NDW) $c_{J,N+1}$ and $w_{j,N+1}$, using the equations [45]

(4) $${\hat{w}}_{j,N+1}=\overset{r_{j}}{\underset{k=1}{\sum }}a_{j,k}w_{j,2^{j}\left(k-1\right)},\;\;{\hat{c}}_{j,N+1}=\overset{r_{J+1}}{\underset{k=1}{\sum }}a_{J+1,k}c_{J,N-2^{J}\left(k-1\right)}.$$

Therefore, the prediction equation will be written as

(5) $${\hat{X}}_{N+1}=D_{N}^{T}\alpha$$

where

- *T* signifies the transposition,
- *D_N_* is formed by the dyadic lagged coefficients contained in the vectors *w* and *c^T^*,
- α is the vector of the unknown parameters, determined as
  (6) $${\hat{\alpha }}_{N}=\underset{\;\;\;\;\;\;\;\alpha }{\arg\;\min}\sum_{k=1}^{r_{J+1}}{\left(X_{k}-D_{k-1}^{T}\alpha \right)}^{2},$$
  and *M* is a fixed integer.

Two other vectors are involved in finding the solution of (5):

- *c*, which contains the coefficients $c_{J,N+1}$,
- *w*, which contains the weights in the linear combination of the actual versus the previous variables’ values.
- Therefore, the procedure has the following stages [45].
- Perform the NDTW using Haar wavelets.
- For a sequence from a time series (*X*_1_, ..., *X_N_*) at a level *J*, the output will be formed of (*J* + 1) vectors of size *N*.
- Build the Equation (5).
- For this aim, at all the decomposition levels, the maximum number of lagged predictor variables is set. Denote them by ($r_{j}^{\left(1\right)}$, …, $r_{J}^{\left(1\right)}$). $\;r_{j}^{\left(1\right)}$ (*j* = 1, …, *J*) is defined to be “the order of the AR process fitted on the dyadically downsampled version of *W_j_* (or *C_J_*) starting from the last coefficient”.
- Estimate Equation (5) utilizing the stepwise regression that links *X_t_* and *D_t_*_−1_.
- Compute the prediction using Equation (5).

#### 2.2.4. Wavelet-ANN Model

A wavelet-artificial neural network (WANN) is obtained by combining a feed-forward neural network with the wavelet model. Given that WANNs permit an efficient selection of the network input, can capture the nonlinearities, and converge to the global minimum objective function, they have been successfully utilized in various applications from atmospheric sciences, hydrology, and economics for short and long term forecast [46,47,48,49,50,51,52].

**Figure 4:**
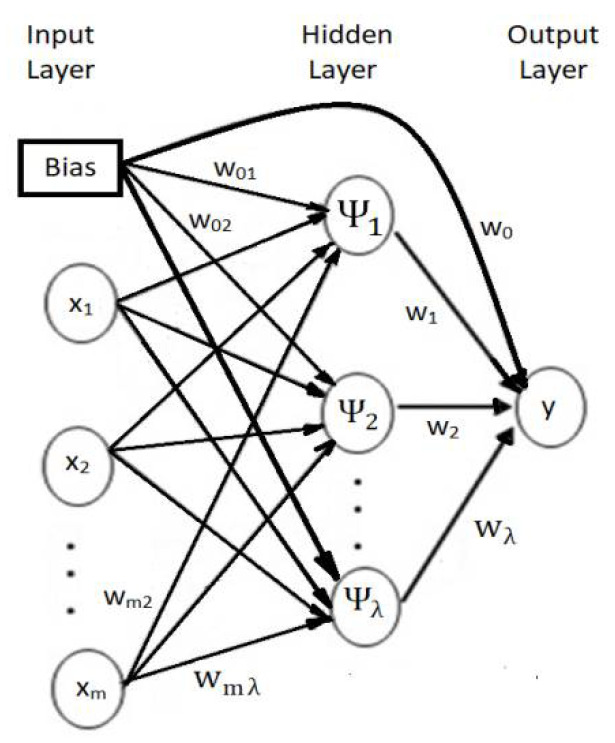
Wavelet-ANN ($x_{1},\;\ldots ,\;x_{m}$ are the input values).

In this study, a WANN with the following layers has been utilized (Figure 4) [31].

- Input layer, where the regressors’ values (lagged variables) are introduced.
- Hidden layer, formed by wavelons, instead of neurons. The variables coming from the previous layer are firstly transformed and decomposed in this layer. Then, the network processes separately each part, utilizing as activation functions (logistic, in this case) elements of an orthonormal wavelet basis.
- In this study, the maximal overlap discrete wavelet transform (MODWT) and the Morlet function have been utilized. For details, the reader may see in [31].
- Output, which estimates the recorded (target) values.

The number of parameters in the WANN from Figure 4 is the *pq* + 2*q* + 1, where *p* = the lag, and *q* = the number of nodes in the hidden layer.

For running the algorithm, the maximum decomposition level was determined to be 6. The number of lags was varied from 1 to 12. The best result, reported here, was obtained with lag = 5.

## 3. Results

**Figure 5:**
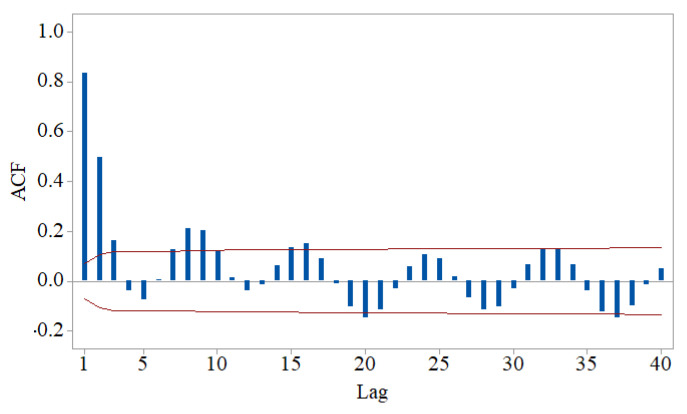
Charts of the signal autocorrelation function (ACF) (obtained using Minitab17).

The Shapiro–Wilk and the runs test rejected the normality and randomness hypotheses, respectively. The KPSS and Levene tests did not reject the stationarity and homoskedasticity hypotheses. The autocorrelation function (ACF) chart from Figure 5 confirms the signal’s autocorrelation.

As the stationarity hypothesis cannot be rejected, and ACF has a decreasing damped sinusoid form, one may search for an ARMA(*p,q*) model with *p* > *q*. The best model found by applying Box–Jenkins methodology was of ARMA(3,1) type, with the coefficients (standard errors, respectively):

*φ*_1_ = 2.115 (0.075), *φ*_2_ = −1.754 (0.103), *φ*_3_ = 0.581 (0.047), and *θ*_1_ = −0.648 (0.084),

AIC = 2951.47 and SCH = 2974.82.

Therefore, the models’ equation is

(7) $$\;\;\;\;\;\;X_{t}=\varepsilon_{t}+2.115\;X_{t-1}-1.754X_{t-2}+0.581X_{t-3}-0.648\varepsilon_{t-1}$$

After applying the Student *t*-test, it was found that the coefficients are significant at 0.001. The Box–Ljung test led to the rejection of the residuals’ autocorrelation. The Shapiro–Wilk and Levene test failed to reject the null hypotheses of normality and homoskedasticity, respectively, for the residual series. Thus, model (7) is correct from a statistical viewpoint.

**Figure 6:**
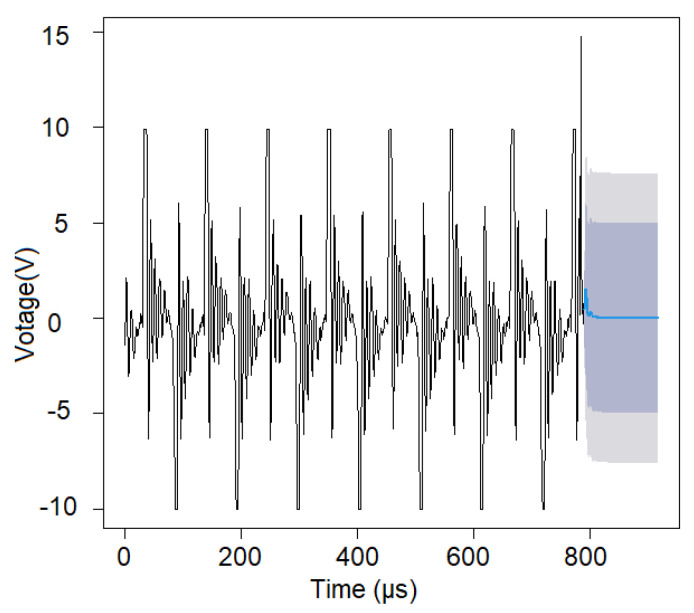
The signal (black) and forecast (blue) and the 0.95 and 0.99 confidence levels (at the right-hand side in different nuances of grey) (obtained using the R software).

The model was used to predict 48 values. These values are plotted in Figure 6 (blue), after the recorded ones (black), accompanied by the confidence intervals at 0.95 and 0.99 (in grey in Figure 6). The patterns of the signal and forecast are not similar, so the model should be improved. Therefore, the GRNN was built as described in Section 2.2.2.

**Figure 7:**
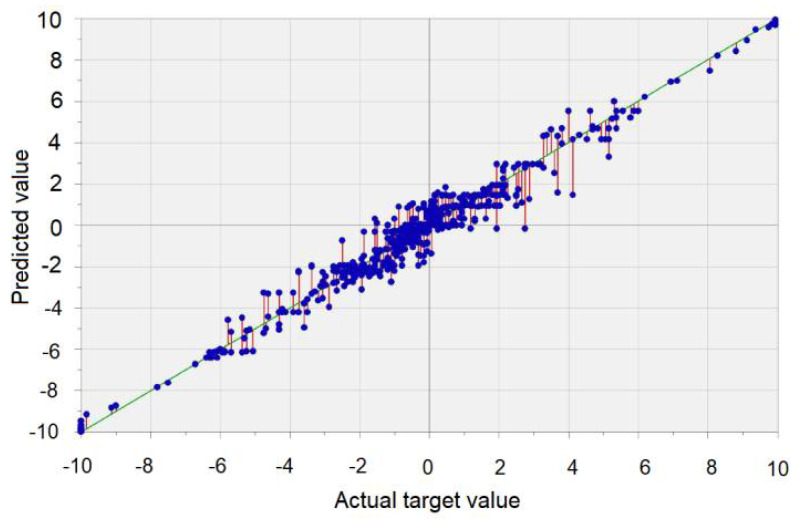
Predicted values (computed by the algorithm) vs. the recorded ones (voltage-V) (obtained using the DTREG software).

Figure 7 contains the plot of the values computed by the algorithm vs. those recorded. Remark the display of the points cloud along the green line representing the ideal situation of the superposition of the recorded and computed values, indicating a good fit.

**Figure 8:**
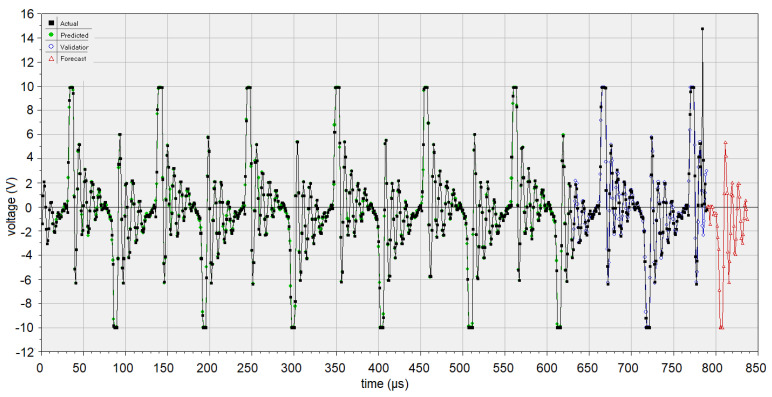
GRNN model. The *Actual* (recorded data), *Predicted* (computed values on the training set), *Validation* (computed values on the test set), and *Forecast* (the new data series built using the GRNN model) sets (obtained using the DTREG software).

Using the trained network, the forecast of 48 values (unknown) of the data series is done. The pattern of subseries built by these new values is similar to that of the initial signal (Figure 8, the red curve), confirming the model performances (discussed in the next section).

**Figure 9:**
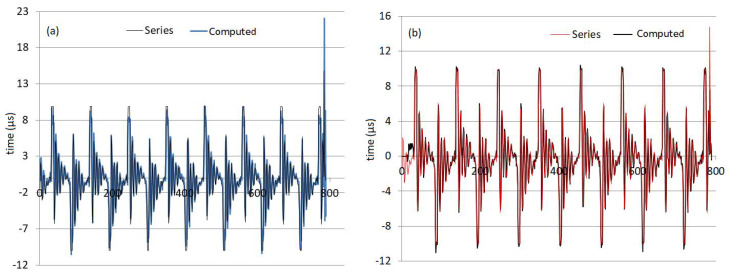
The recorded and computed values of the signal in (**a**) Wavelet-ARIMA and (**b**) Wavelet-ANN model (output from the R software).

The charts of the recorded and computed values of the signal by using the hybrid algorithms are presented in Figure 9. Comparing Figure 9a,b, one may remark that the Wavelet-ARIMA model generally underestimates the maximum recorded values. The Wavelet-ANN model does not fit the first ten series values well but better fits the rest of the data series.

**Figure 10:**
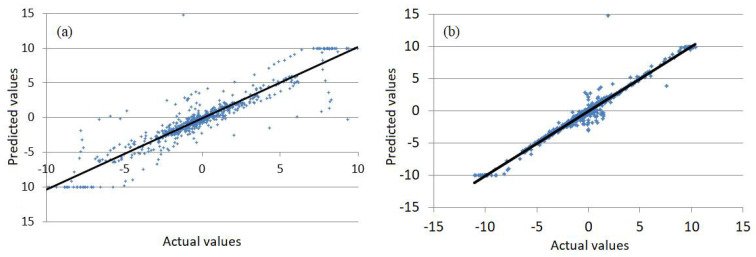
Computed values (Predicted) versus the recorded ones (Actual) in (**a**) the Wavelet-ARIMA model) and (**b**) the Wavelet-ANN model (output from the R software).

Figure 10 emphasizes the best forecast quality of the Wavelet-ANN model. It contains the chart of the computed values plotted against the recorded values. The points that have the coordinates (actual, predicted) are situated along the first bisectrice of the coordinates axes in both Figure 10a,b. Still, they are more dissipated in Figure 10a, corresponding to the Wavelet-ARIMA model, than in Figure 10b, corresponding to the Wavelet-ANN model.

**Figure 11:**
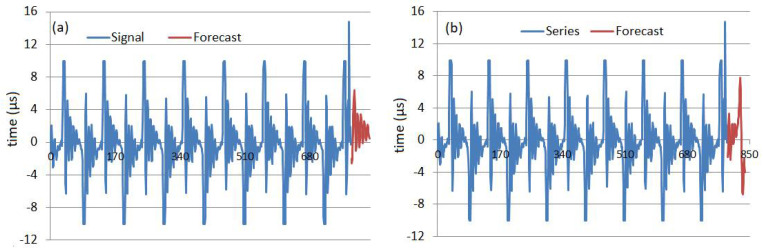
The signal (blue) and the forecast (red) in (**a**) the Wavelet-ARIMA and (**b**) Wavelet-ANN model (red).

The charts of the signal and forecast of the next 48 values obtained using the Wavelet-ARIMA and Wavelet-ANN are presented in Figure 11.

The Wavelet-ARIMA forecast series (in red, Figure 10a) is sinusoid decreasing. The Wavelet-ANN forecast series (in red, Figure 10b) and the signal have the same shape. Comparison of the forecast obtained by GRNN and Wavelet-ARIMA models show the same shapes and the highest amplitude for the last signal.

## 4. Discussion

To compare the models’ performances, we analyze the quality indicators.

For the ARIMA(3,1) model, the mean standard error (MSE) = 2.4278 and the mean absolute error (MAE) = 0.7747. Mean absolute predicted error (MAPE) could not be computed because there were some values equal to zero, so the formula did not permit the division by zero.

The indicators of the GRNN model’s quality are listed in Table 1, together with their values.

On the training set, the variation explained by the (R^2^) and correlation between actual and predicted errors (r_ap_) are very high, the mean standard error (MSE) and mean absolute error (MAE) are small, while the mean absolute predicted error (MAPE) is 18.43%, indicating a good behavior of the algorithm on the training set. On the test set, the values of R^2^ and r_ap_ are smaller than on the training set, and MAE and MSE remain low. A significant increase in MAPE is noticed (to 71.577%).

After using the hybrid approaches, the values computed for the goodness of fit indicators on the test sets are

- for the Wavelet-ARIMA: R^2^ = 82.4829%, MAE = 0.858, MSE = 2.656, MAPE (%) = 98.81, r_ap_ = 0.907
- for the Wavelets-ANN: R^2^ = 96.74%, MAE = 0.300, MSE = 0.489, MAPE (%) = 42.76, r_ap_ = 0.984.

Comparing the performances of the artificial intelligence-based models on the test sets (relevant for how well the model learns the data and can use it), the best model is WANN, no matter what indicator is considered. This is confirmed by comparing Figure 8 and Figure 11 that show the forecast for 48 μs.

ARIMA models have been widely used in real-life problems modeling given their ability to capture the linear dynamics of the phenomena. Still, as many processes present nonlinear patterns, the use of ARIMA is not always the best choice (given that it assumes the existence of linear correlation in the data series) [31]. The main advantages of ANNs are their flexibility in modeling nonlinear features of data series, and the absence of the constrain on the a priori specifying the model form [52]. The methods relying on the on wavelet transform can well describe the signal’s multiscale features [51]. Combining these algorithms will benefit all their properties.

The results of this study are consistent with the findings of other researchers that emphasized that the use of hybrid approaches may improve the models’ performances [31,32,51,52,53].

## 5. Conclusions

The statistical analysis of the electrical signal induced by ultrasound cavitation in seawater showed that the signal is not random, is stationary and homoskedastic. An ARMA model has been proposed and validated from a statistical viewpoint based on these characteristics. As the ARMA model did not provide a good forecast of the following 48 values, alternative models have been proposed and compared to find the best alternative. The first one—the GRNN model—learned the data well, gave good results on the test set, and was successfully employed for forecasting the next (unknown) 48 values of the series. The last two models were of the hybrid type.

The Wavelet—ARIMA model improved the forecast by comparison with the ARMA one; still, R^2^ = 82.4829%, and MAPE = 98.81% (high enough). Considering that the lowest the MAPE is, the better the model is, the best model was the Wavelet-ANN one (with R^2^ = 96.74%, and MAPE (%) = 42.76).

The study provided for the first time such modeling and comparison for the signal collected in seawater, in the cavitation field, using the experimental setup built by our team and built four alternative models, the hybrid ones being able to provide good forecasts. Similar analyses will be performed to verify that the last approach is the best one for modeling the signals collected in the same or different liquids (diesel, tape water, and transformer oil) to other powers of the ultrasound generator. These liquids were selected because our research team has published some preliminary results and until now good models for some of them were not obtained yet. The most important feature that recommends this hybrid model is its flexibility to capture the series’ nonlinearities. This procedure has no limitation related to the liquid type.

## Figures and Tables

**Table 1:** Indicators of the GRNN model’s quality.

Indicator	Training Set	Test Set
R^2^	98.97%	85.44%
MSE	0.150	2.312
MAE	0.133	0.474
MAPE(%)	18.423	71.577
r_ap_	0.995	0.925

## Data Availability

Data will be available on request from the authors.
